# Subnational Burden of Disease According to the Sociodemographic Index in South Korea

**DOI:** 10.3390/ijerph17165788

**Published:** 2020-08-10

**Authors:** Dun-Sol Go, Young-Eun Kim, Seok-Jun Yoon

**Affiliations:** 1 Department of Health Care Policy Research, Korea Institute for Health and Social Affairs, Sejong 30147, Korea; kds237@gmail.com; 2 Big Data Department, National Health Insurance Service, Wonju 26464, Korea; kimyes4454@gmail.com; 3 Department of Preventive Medicine, Korea University College of Medicine, Seoul 02841, Korea

**Keywords:** social determinants of health, population health, burden of disease, sociodemographic index, disability-adjusted life years, South Korea

## Abstract

The sociodemographic index (SDI), a composite index per capita income, educational attainment, and total fertility rate in a country, can indicate whether the country’s burden of disease varies depending upon its level of socioeconomic development. This study identified the subnational SDI and disease burden of South Korea based on the country’s overall SDI, using national representative data. The burden of disease was measured using disability-adjusted life years (DALY) with an incidence-based approach. We used National Health Insurance Services claims data to estimate the years lived with disability (YLD) and cause-of-death statistics to estimate the years of life lost (YLL). Indicators of subnational SDI were also extracted. The Korean subnational SDIs for 250 regions were correlated with YLL, YLD, and DALY for the year 2016. The correlation between SDI and YLL was stronger in big cities than in medium areas and small areas. Moreover, the higher the SDI, the higher the coefficient. The SDI should be used as a standard for interpreting and comparing regions’ disease burden at the subnational level.

## 1. Introduction

Health differences among individuals within a population and among populations themselves are affected by regional factors [1]. Identifying the determinants of health, not only at the individual level but also at the regional level, is essential for planning policies aimed at eradicating those differences. The Korean National Burden of Disease Study measured the subnational burden of disease by using the disability-adjusted life years (DALY) metric for 250 regions in Korea [2,3]. To identify and interpret a region’s ranking, the regions were compared with adjacent regions based only on the location, regardless of its other characteristics. However, the degree of socioeconomic development of each region, as a metric, would aid in making a more reasonable comparison.

To verify whether a country’s burden of disease varies according to degree of socioeconomic development, the Global Burden of Disease Study (GBD) developed the Sociodemographic Index (SDI) [4]. The SDI is a summary measure that quantifies the level of sociodemographic development; it is the composite average of the rankings of per capita income, average educational attainment, and total fertility rate and is expressed on a scale from 0 to 1. The national SDI of South Korea was 0.87 in 2017, based on a GBD of 195 countries. The country’s SDI has increased from 0.60 in 1980 to 0.87 in 2017, and has been 0.85 or higher since the year 2009 [4]. Despite this high level of SDI compared to other countries, it is necessary to identify the status of sociodemographic development at the subnational level in South Korea.

The SDI has a strong correlation with health outcome variables such as mortality, life expectancy, and DALY, and is used as a basis for predicting health outcomes and comparing regional health outcomes. Globally, life expectancy at birth is rising; however, the difference between high-SDI countries and low-SDI countries has increased from 4.7 in 1950 to 5.1 in 2017 [5]. In addition, although the difference in life expectancy between men and women has decreased since 1990 in high-SDI countries, middle, low-middle, and low-SDI countries have only witnessed a decrease since 2010. The GBD suggests that policy makers in countries that may experience changes in disease burden characteristics because of epidemiological transition should evaluate the effectiveness of policies by comparing international health outcomes with the health outcomes of sociodemographic development alone [4].

This study aimed to estimate the SDI at the subnational level and verify its use in interpreting the subnational burden of disease in South Korea.

## 2. Materials and Methods

We included 250 municipal-level administrative districts in South Korea called *si, gun*, and *gu*. The administrative districts comprise 67 cities (“*si*” in Korean denotes a city with a population of at least 50,000), 114 counties (“*gun*” in Korean denotes a county with a population of less than 50,000), and 69 districts (“*gu*” in Korean denotes a district with a relatively smaller population).

The SDI was computed as the geometric mean of per capita income, average educational attainment, and total fertility rate. These three components were rescaled by min–max standardization. The per capita income data were extracted from the Korea Community Health Survey, which is a nationwide, community-based, cross-sectional survey conducted by the Korea Centers for Disease Control and Prevention, annually since 2008 [6]. We used the data from 2016. The monthly average household income for one year was divided by the square root of the household size. The questionnaire regarding the average monthly income of the households had eight response categories (less than USD 428, USD 428–857, USD 858–1714, USD 1715–2571, USD 2572–3428, USD 3429–4284, USD 4285–5140, and more than USD 5141; where USD 1 = KRW 1167, based on the exchange rate on January 3, 2020). The median value of each category was converted into the monthly household income of respondents and the category “more than USD 5141” was converted to USD 5570 so that the average income of the survey respondents was similar to the national average in 2016 (USD 2184). Data regarding educational status were obtained from the Population and Housing Census. For example, in the case of individuals who had dropped out of middle school, the median was calculated to be 7 years (minimum 6 years and maximum 8 years). The fertility rate under 25 years of age was measured by the ratio of the annual number of births to women aged between 15 and 25 years, per 1000 women in that age group. The total birth rate data by age and district were obtained from Internal Migration Statistics.

The DALY was measured using the incidence-based approach. The estimation was based on the Korean National Burden of Disease Study, which is contextualized to the Korean population of the GBD study based on a modified disease classification and country-specific data sources [2,7]. Years of life lost (YLL)—which refers to premature death within a population—were calculated by subtracting the age at death from the life expectancy at that age. The gender- and age-stratified cause-specific death data were obtained from Statistics Korea. Years lived with disability (YLD)—which refers to the loss of healthy years due to morbidity—was calculated by measuring the incidence of disease and disability weight. The epidemiological data for each disease were drawn from the Korean National Health Insurance Service data, a representative medical-use database covering approximately 98% of the population. Disability weight was obtained from a survey of the national population [8]. To standardize health outcome metrics by the regional population structure, we used the direct age standardization method with the mid-year population of South Korea in 2005.

To verify whether the SDI could be used as a standard for comparing the region-specific burden of disease in South Korea, we determined the correlation between the SDI and each health outcome at 250 subnational levels. Pearson’s correlation coefficient with a significance level within 5% was used. All statistical analyses were performed using SAS (Version 9.4, The SAS Institute, Cary, NC, USA).

The study protocol was approved by Korea University’s institutional review board (KU-IRB-18-EX-51-A-1). This study used data that do not contain personal information. The data were provided by the review of National Health Information data request review committee (NHIS-2019-1-182).

## 3. Results

**Figure 1:**
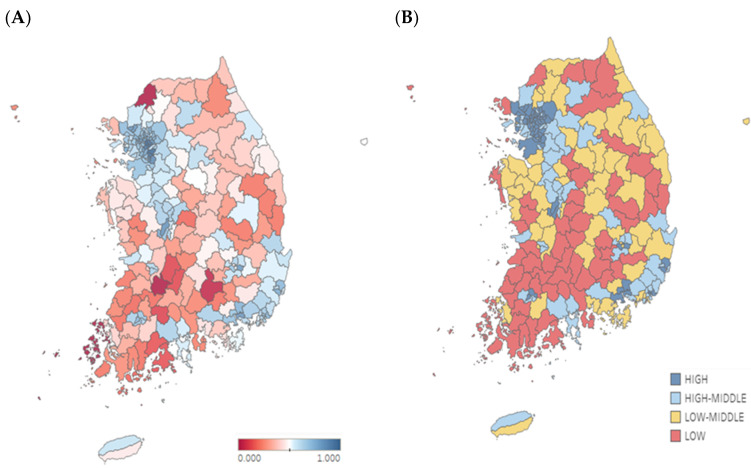
Subnational SDI in Korea. (**A**) SDI by 250 regions, (**B**) SDI by quartile. SDI—Sociodemographic index.

The Korean subnational SDIs in 2016 are shown in Figure 1A. The average of the per capita income was USD 1582 (KRW 1.86 million), with a maximum of USD 3036 (KRW 3.57 million) in Gangnam-gu and a minimum of USD 689 (KRW 0.81 million) in Sinan-gun. The average educational attainment for the population above 15 years of age was 10.9 years. The region with the longest educational attainment was Seocho-gu (14.8 years) and that with the shortest educational attainment was Imsil-gun (7.9 years). The fertility rate under 25 years of age was 8.23 per 1000 women on average. Yeoncheon-gun had the highest fertility rate (20.71 per 1000 women), while Gwacheon-si had the lowest fertility rate (0.86 per 1000 women). To summarize, the 250 regions could be grouped by the SDI quartiles: high, high-middle, low-middle, and low. Figure 1B shows the SDI quartile groups.

**Figure 2:**
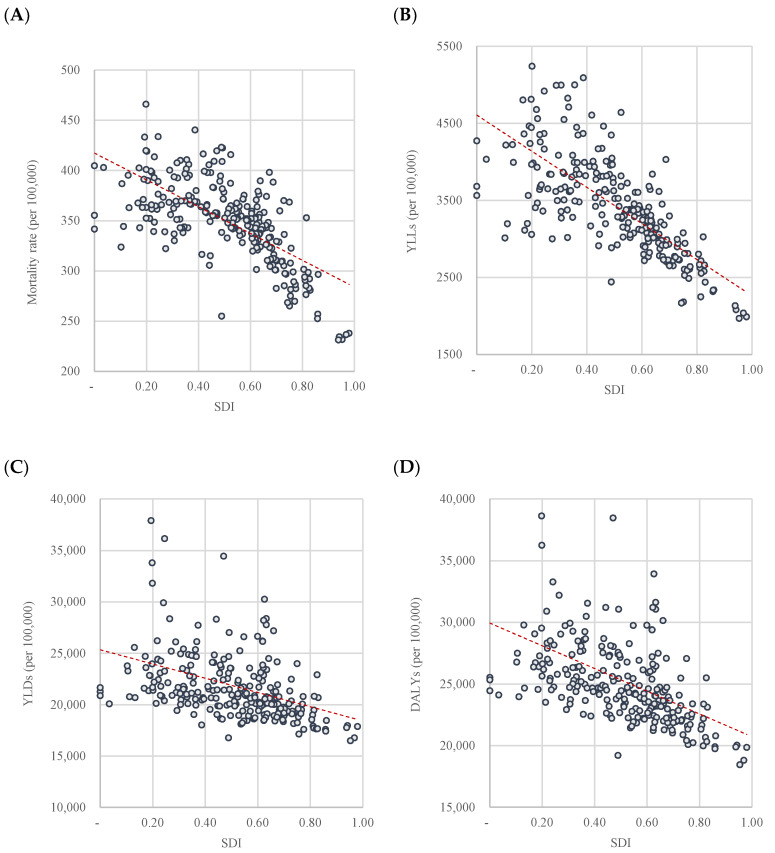
Correlation between SDI and disease burden. (**A**) Mortality rate. (**B**) YLL. (**C**) YLD. (**D**) DALY. SDI—sociodemographic index; YLLs—years of life lost; YLDs—years lived with disability; DALYs—disability-adjusted life years.

The Korean subnational SDIs for 250 regions were statistically correlated with health outcome measures (Figure 2). The higher the SDI, the lower the burden of disease metrics. The correlation coefficient was greatest for YLL (r = −0.736, *p* < 0.0001; Figure 2B), followed by mortality rate (*r* = −0.673, *p* < 0.0001; Figure 2A), YLD (*r* = −0.362, *p* < 0.0001; Figure 2C), and DALY (*r* = −0.459, *p* < 0.0001; Figure 2D). 

**Figure 3:**
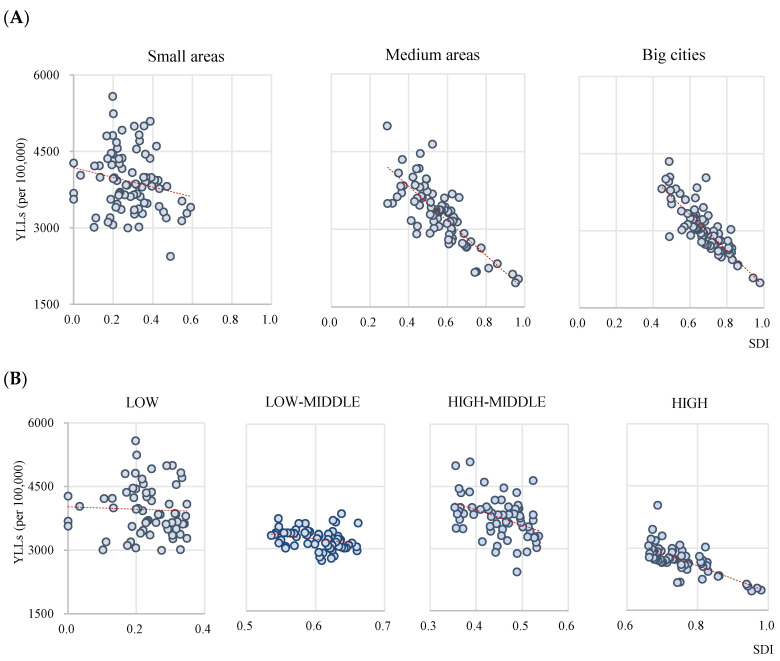
Correlation between SDI and YLL by subgroups. (**A**) Regions. (**B**) SDI quartiles. YLLs—years of life lost; SDI—Sociodemographic index.

Figure 3 illustrates the correlation between the SDI and YLL by subgroups. We classified the 250 regions by the population of those regions: big cities, if they had a population of over one million; medium areas, if the population of the “si” and “gu” combined comprise less than one million; small areas with the other “gun”. The correlation between the SDI and YLL was found to be stronger in big cities (*r* = −0.827, *p* < 0.0001) than in medium areas (*r* = −0.802, *p* < 0.0001), while small areas showed no statistically significant correlation (*r* = −0.197, *p* = 0.008; Figure 3A). According to the SDI quartile groups (Figure 3B), the correlation between the SDI and YLL was stronger in the high group (*r* = −0.720, *p* < 0.0001) than in the high-middle (*r* = −0.405, *p* = 0.001) and low-middle group (*r* = −0.269, *p* = 0.033). In the low group, the coefficient was not statistically significant (*r* = −0.720, *p* < 0.0001).

## 4. Discussion

This study measured the SDI at the subnational level in South Korea in 2016, using country-contextualized data sources. We adopted the SDI metric at the subnational level in South Korea for 250 administrative regions. Although the country is globally ranked 20th out of 195 countries in terms of national development status and is included in the high SDI group, we confirmed disparities in SDI at the subnational level. In Japan, the subnational difference in SDI was 0.072 (Tokyo, 0.920; Okinawa, 0.848) in 2016, which is the difference in Korean SDI between 1999 and 2016. The “region” reflects the “space of the risk,” in that individuals and populations in the same space are affected by similar health determinants. Identifying regional health status allows us to understand the needs that health policies must address.

To verify whether the SDI could be used as a standard for analyzing the subnational disease burden, we analyzed the correlation between the SDI and the burden of disease metrics (mortality, YLL, YLD, and DALY). The results showed that the higher the SDI, the lower the burden of disease metrics. The statistically significant correlation between the SDI and disease burden implies that the SDI is a useful substitute for health outcomes at the subnational level in South Korea. In addition, the relation between the SDI and disease burden is in line with international previous studies using the SDI to compare the health status of countries [9,10,11]. Meanwhile, national previous studies have addressed the relations between individual sociodemographic indicators such as income, educational status, occupational status, and health at regional levels in South Korea [12,13]. Compared to these individual indicators, the composite index has advantages in summarizing the multidimensional reality, especially in terms of supporting policy making. In addition, health-related composite indexes have been used to interpret the regional health status [14,15]. However, most of them include health indicators, which should be excluded when using an index to compare health. Even though the deprivation index has been used to interpret regional health in order to reflect regional sociodemographic status, the correlation between SDI and health outcomes in this study (*r* = −0.673, *p* < 0.0001) was greater than the coefficients reported in previous studies of the health determinants index and health outcomes.

We also confirmed that the SDI better describes the health outcomes related to mortality than does morbidity. The correlation coefficient was higher for YLL than for mortality rate, which implies that the burden of disease due to premature death is greater according to the SDI compared to mortality. However, it was relatively weakly correlated with YLD and DALY, which represents disability. Although previous studies have not analyzed the health determinants of disease burden with YLL and YLD, it is known that there are significant correlations between health determinants and self-rated health (*p* < 0.01) [14]. However, the correlation coefficient was less than 0.5, indicating that the ranking of health determinants could not be interpreted as a ranking of health outcomes. When comparing the correlation between the SDI and health outcomes by GBD countries, the correlations for life expectancy and DALY were lower than that of mortality [4,5]. This is because the health determinants affecting mortality and morbidity are different. Further research is required to identify and distinguish among the different sociodemographic factors that affect health differences [13].

The correlations between SDI and health outcomes were stronger in big cities than in medium and small areas. The deprivation index—which was developed in a previous study [16]—is composed of socioeconomic indicators and shows a stronger correlation with mortality rates in big cities than in small areas in South Korea [17]. Jordan et al. reported that in small areas [18], the vulnerable population is spread out, compared to in cities, which makes accurate measurement difficult. To supplement this, it is necessary to develop indexes with separate indicators for cities and small areas or to consider spatial correlation [19].

This study has some limitations. The study adopted the SDI by considering only three components: per capita income, education, and fertility rates. The GBD had to estimate the SDI for 195 countries; therefore, it would have considered indicators that were both available and easier to interpret. However, in South Korea, as the differences in health outcomes by educational level and fertility rate decline, other health determinants—such as employment status and resources—become important. Further, the SDI’s use is limited to factors that explain the level of development or health outcomes. Thus, an index that includes health indicators should be developed to better explain the health differences among Koreans [3]. The data source for educational attainment and the Population and Housing Census cover 20% of the total South Korean population. While this is sufficient to represent the entire population, there may still be limitations in using a sample. Nevertheless, the Population and Housing Census is the only source of nationally accredited statistics that can estimate the educational level of South Koreans representatively. As a cross-sectional study, the current results are limited to only the study period, the year 2016. Therefore, longitudinal studies need to identify aspects of community health status and health determinants more broadly.

The estimation and monitoring of health status at the subnational level is essential for identifying health differences and establishing appropriate policies. The SDI can be used to summarize complex and multidimensional realities from the perspective of supporting policy decisions, and is incredibly useful for comparing disease burdens among countries and regions. Even in regions with the same SDI value, the indicators that comprise the index may be different; this raises the question of whether it is reasonable to view the regions as having the same characteristics [18,20,21]. Nevertheless, assessing the SDI at the subnational level is meaningful, as it presents a standard of reference for estimating health status using health determinants as composite indicators.

## 5. Conclusions

This study measured the Korean subnational disease burden by using the DALY metric and suggested the use of the SDI to compare subnational disease burden. The SDI can be used to track progress in key areas of socioeconomic development, and monitor inequalities across and within nations. Governments can use the index to predict health outcomes and identify areas that need additional support. Researchers can also use the index as a control variable when examining health outcomes and analyzing the impact of health systems on health outcomes.
